# Pneumococcal Sepsis Complicated by Splenic Abscesses and Purpura Fulminans in a 15-Month-Old Child: Case Report and Review of the Literature

**DOI:** 10.1177/2324709616636398

**Published:** 2016-02-29

**Authors:** Scott Pangonis, Pisespong Patamasucon, Ellen Fitzpatrick

**Affiliations:** 1 University of Nevada School of Medicine, Las Vegas, NV, USA; 2 Sunrise Children’s Hospital, Las Vegas, NV, USA

**Keywords:** *Streptococcus pneumoniae*, splenic abscess, purpura fulminans

## Abstract

*Streptococcus pneumoniae* is an invasive organism that causes a wide range of common diseases, including sinusitis, acute otitis media, and pneumonia. Splenic abscesses and purpura fulminans (PF) are rare complications of pneumococcal disease. Splenic abscesses caused by *S pneumoniae* have only been reported in the adult literature. PF has been described in the pediatric population as a rare complication in patients with invasive pneumococcal disease (IPD) with and without underlying immunological disorders such as asplenia. Here, we report a patient with IPD complicated by splenic abscesses and PF. Our patient initially presented with bacteremia, septic shock, and disseminated intravascular coagulation. She subsequently developed PF and splenic abscesses. She survived her illness after receiving a total of 8 weeks of antibiotic therapy. This case highlights 2 rare complications of IPD and demonstrates the need to keep pneumococcal disease in the differential diagnosis even in children whose vaccination status is up to date.

## Introduction

Invasive pneumococcal disease (IPD) is deﬁned as the recovery of an isolate of *S pneumoniae* from a normally sterile site, such as blood, cerebrospinal ﬂuid (CSF), pleural ﬂuid, joint aspirate, pericardial ﬂuid, or peritoneal ﬂuid.^1^ Splenic abscesses are rare in the pediatric population.^2^ Early recognition and intervention are critical because of the high mortality rate associated with delayed diagnosis.^3^ Splenic abscesses and purpura fulminans (PF) are rare complications of IPD. Splenic abscesses caused by IPD have been reported in adults.^4^ We report a case of IPD confirmed by blood culture and serotyping in a fully vaccinated 15-month-old girl whose clinical course was complicated by splenic abscesses and PF. To our knowledge, this is the first case of splenic abscesses caused by *Streptococcus pneumoniae* described in the pediatric population.

## Case Report

This patient was a 15-month-old girl who presented to a local emergency department with fever and cough for 1 day. She was diagnosed with a viral upper-respiratory infection and discharged home. She subsequently became lethargic with poor oral intake over the next day, which prompted her parents to return to the emergency department. She was healthy prior to this illness. Her childhood vaccinations were up to date, including 4 doses of the 13-valent pneumococcal conjugate vaccine (PCV-13). Her family history was negative for immunodeficiencies and sickle cell disease.

**Figure 1.**
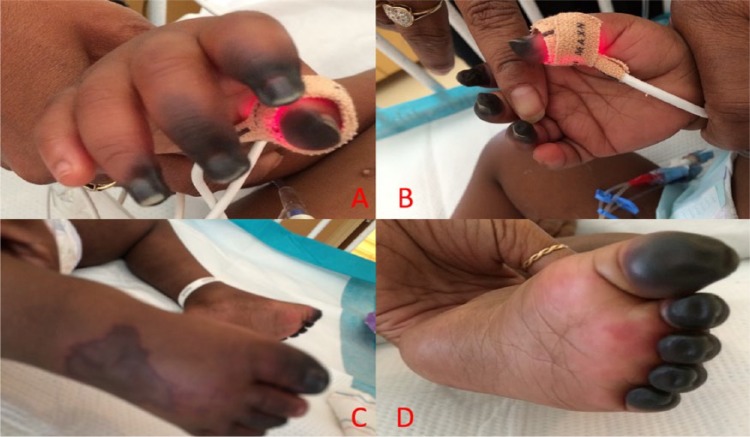
Digital infarctions of the distal phalanges of the right upper extremity (A and B) and the lower extremities (C and D).

**Figure 2.**
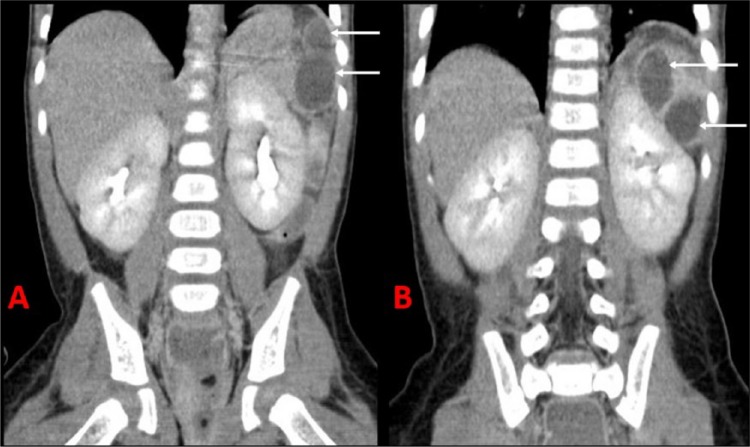
Computed tomography scan of the abdomen revealing multiple splenic abscesses (demonstrated by arrows) in the coronal views from hospital day 9 (A) and hospital day 41 (B).

On arrival, she was found to have a temperature of 40.9°C, heart rate of 184 beats/min, respiratory rate of 36 breaths/min, and blood pressure of 53/26 mm Hg. Physical exam revealed a toxic-appearing toddler. She was tachycardic and tachypneic, with delayed capillary refill. Her abdominal exam on admission revealed no organomegaly. Physical exam was otherwise noncontributory. Laboratory investigations revealed a pH of 7.1; PCO_2_ of 35 mm Hg; PO_2_ of 52 mm Hg; HCO_3_ of 11 mg/dL; prothrombin time of 37.8 s; international normalized ratio of 3.8; activated partial prothrombin time of 101.4 s; total leukocyte count of 1900/mm^3^, with 10% banded neutrophils and 25% segmented neutrophils; hemoglobin of 10.8 g/dL; and platelet count of 67 000/mm^3^. Lumbar puncture was performed and revealed a white blood cell count of 1 per mm^3^, red blood cell count of 3 per mm^3^, glucose 108 mg/dL, and protein 83 mg/dL. Blood, urine, and CSF cultures were sent, and she was started on broad spectrum antibiotics with vancomycin and ceftriaxone. She was admitted to the pediatric intensive care unit for management of septic shock, disseminated intravascular coagulopathy, and multiple organ dysfunction syndrome. The blood culture from admission became positive after 8 hours of growth, which was identified as *S pneumoniae* that was sensitive to penicillin and ceftriaxone, with a minimum inhibitory concentration of less than 0.03 µg/mL. An immunological workup revealed a normal antibody response to childhood vaccinations, including diphtheria, tetanus, and pneumococcus; IgM was 82 mg/dL, IgG was 986 mg/dL, and IgA was 106 mg/dL; total complement (CH50) was 63 U/mL, and alternative complement pathway (AH50) was 65%, all of which were within normal limits. *S pneumoniae* IgG to 14 serotypes from ARUP laboratories demonstrated >1.3 µg/mL for 10 of the 14 serotypes, which is consistent with an adequate vaccination response. Newborn screen was negative for sickle cell disease, and there was no family history of sickle cell disease. Vancomycin was discontinued after reviewing the sensitivities and documentation of sterile cerebrospinal fluid. She was initially hypotensive despite being placed on vasoactive medications for treatment of refractory shock. On hospital day 4, she developed dry gangrene of the distal phalanges (Figure 1). On hospital day 8, she remained febrile after resolution of septic shock. Subsequent physical exam revealed a tender mass 3 cm below the left costal margin, which prompted an evaluation. A computed tomography (CT) scan of the abdomen with contrast obtained on hospital day 9 revealed 3 rim-enhancing splenic abscesses measuring 3.4, 2.4, and 1.4 cm (Figure 2A). Our pediatric infectious disease service was consulted for assistance with the management of splenic abscesses, and they suggested consulting pediatric surgery for surgical intervention in addition to antibiotic therapy. Transthoracic echocardiogram on day 11 of hospitalization did not reveal intracardiac vegetations. MRI of the brain was only significant for mild atrophy and did not reveal any brain abscesses. Pediatric surgery and interventional radiology were consulted, and both recommended medical management to avoid potential complications—that is, bleeding and spread of infection into the pleural space. Clindamycin was added because of its penetration into abscess fluid, although susceptibilities were not performed by the microbiology laboratory. The pathogen isolated from the initial blood culture was sent to the Nevada State Public Health Laboratory for serotyping, which revealed the presence of serotype 22F/A. After discussion with Hematology, an evaluation for an underlying coagulopathy was not performed because her coagulation studies normalized after treatment of her infectious process. All repeat blood cultures revealed no growth of bacterial pathogens. The patient’s splenic abscesses were monitored with weekly ultrasounds. She completed a 14-day course of intravenous ceftriaxone for treatment of *S pneumoniae* bacteremia. Antibiotics were narrowed to intravenous penicillin G potassium to complete her treatment course for her splenic abscesses. Repeat CT scan of the abdomen on hospital day 41 revealed a decreased size of the splenic abscesses, with the largest collection measuring 1.5 cm; resolution of the smallest collection; and lack of rim enhancement (Figure 2B). The patient demonstrated an allergy to penicillin G and cephalexin at the end of her treatment course. Therefore, it was decided to change to clindamycin even though there was no documented susceptibility, and this was continued for an additional 2 weeks. Repeat ultrasound 2 weeks after discharge demonstrated resolution of all but 1 splenic abscess, which was now 0.9 cm. Antibiotics were discontinued at that time. Her antibiotic course included a total of 6 weeks of IV antibiotics and 2 additional weeks of oral antibiotics. The remaining abscess had resolved as of her 1-month follow-up visit. She underwent debridement after autoamputation of her necrotic digits. Currently, she remains afebrile as of her 6-month follow-up visit.

## Discussion

Splenic abscesses are a rare occurrence in the pediatric population.^5^ The diagnosis of splenic abscess is often not considered in children because of its rarity and nonspecific clinical features.^3^ Early recognition is crucial because the mortality rate in patients with splenic abscesses left untreated can reach up to 100%.^2^ Common findings include fever, left-upper-quadrant or diffuse abdominal pain, splenomegaly, and leukocytosis.^3^ Our patient had splenomegaly, abdominal tenderness, and persistent fevers despite being on adequate antibiotic therapy. The predisposing factors for splenic abscesses include malignant or contagious spread, hematological disorders such as sickle cell disease, immunodeficiency, and direct splenic trauma.^2^ Alcoholism was identified as a risk factor for developing intra-abdominal abscesses in adults with IPD.^4^ There was no history of splenic trauma, and neither was there evidence of immune dysfunction found on laboratory evaluation that would have made her more susceptible to IPD.

According to a review of 67 cases of splenic abscesses by Chang et al,^6^ Gram-negative bacillus infections were more common (55.2%) than those with Gram-positive coccus (31%). Three cases of splenic abscesses caused by *S pneumoniae* were identified in the adult population.^5,7^ One patient had a history of alcoholism and was diagnosed with “Austrian syndrome,” which consists of the triad of endocarditis, meningitis, and pneumonia caused by *S pneumoniae*.^8^ Risk factors were not described for 2 of the patients with splenic abscesses.^4^ *S pneumoniae* was isolated on admission via blood culture after 8 hours of growth, suggesting that our patient’s splenic abscesses were likely a result of direct contiguous spread from bacteremia. A weakness of this case report is that determination of the causative agent of the splenic abscesses was based on a positive blood culture result and not from abscess fluid culture, because the fluid collections were not aspirated and sent for culture. Therefore, the possibility of second bacterial infection or coinfection with a pneumococcal strain that was not 22A/F could not be entirely excluded. However, bacteremia is a known risk factor for developing splenic abscesses, so it was presumed that the 2 processes were related. The fluid collections improved in size while on antibiotic therapy and were no longer rim enhancing at discharge.

Splenic-preserving therapies such as partial splenectomy or interventional radiological guide needle aspiration are now preferred over splenectomy in the pediatric age group to avoid overwhelming postsplenectomy infections.^2^ We elected to treat with prolonged antibiotic therapy to avoid the possibility of these infections. In addition, the presence of multiple abscesses made needle aspiration difficult because this would likely require multiple interventions, increasing the risk of complications such as bleeding or seeding infection into the pleural space. Instead, our patient completed 14 days of intravenous ceftriaxone for treatment of *S pneumoniae* bacteremia and 6 weeks of intravenous penicillin G for the initial treatment of her splenic abscesses. Clindamycin was chosen to complete the patient’s treatment course because the patient developed an allergy to both penicillins and first-generation cephalosporins. Also, sensitivity of *S pneumoniae* to clindamycin has been well documented in the literature among non-19A strains.^9^

Symmetrical peripheral gangrene, also known as PF has been described in the pediatric population as a complication of IPD.^8,10-12^ Most cases of PF caused by *S pneumoniae* infections occur in adults and in patients with asplenia,^8,10^ though cases have been described with no predisposing risk factors.^11,12^ Our patient initially presented with bacteremia, septic shock, disseminated intravascular coagulation, and multiple organ dysfunction syndrome and was later found to have developed digital infarctions as a result of PF and splenic abscesses during her hospitalization. She remained profoundly hypotensive despite vasoconstrictive medications for 10 hours during her initial hospitalization, which would have contributed to her digital infarctions. Our patient did not have any predisposing conditions that would have made her more likely to develop PF, such as sickle cell disease, asplenia, or complement deficiency. She received adequate vaccination against pneumococcus with the 13-valent pneumococcal vaccine, although this does not contain strain 22A/F.

## Conclusion

In conclusion, splenic abscesses are a potential complication in patients with IPD and persistent fevers despite adequate antibiotic coverage. PF is a potential complication of pneumococcal sepsis and should be considered even in children with no underlying immunological disorder. IPD should not be excluded in the differential diagnosis if the child’s vaccination status is up to date because there are many serotypes not covered in the PCV-13.
